# Mobile Sinks Assisted Geographic and Opportunistic Routing Based Interference Avoidance for Underwater Wireless Sensor Network

**DOI:** 10.3390/s18041062

**Published:** 2018-04-02

**Authors:** Farwa Ahmed, Zahid Wadud, Nadeem Javaid, Nabil Alrajeh, Mohamad Souheil Alabed, Umar Qasim

**Affiliations:** 1 COMSATS Institute of Information Technology, Islamabad 44000, Pakistan; farwaahmed17@gmail.com (F.A.); nadeemjavaid@comsats.edu.pk (N.J.); 2 University of Engineering and Technology Peshawar, Peshawar 25000, Pakistan; zahidmufti@nwfpuet.edu.pk; 3 Biomedical Technology Department, CAMS, King Saud University, Riyadh 11633, Saudi Arabia; salabed@ksu.edu.sa; 4 Cameron Library, University of Alberta, Edmonton, AB T6G 2J8, Canada; umar.qasim@ualberta.ca

**Keywords:** opportunistic routing, potential neighbor number, communication void, local maxima, energy consumption, packet delivery, latency, depth adjustment

## Abstract

The distinctive features of acoustic communication channel-like high propagation delay, multi-path fading, quick attenuation of acoustic signal, etc. limit the utilization of underwater wireless sensor networks (UWSNs). The immutable selection of forwarder node leads to dramatic death of node resulting in imbalanced energy depletion and void hole creation. To reduce the probability of void occurrence and imbalance energy dissipation, in this paper, we propose mobility assisted geo-opportunistic routing paradigm based on interference avoidance for UWSNs. The network volume is divided into logical small cubes to reduce the interference and to make more informed routing decisions for efficient energy consumption. Additionally, an optimal number of forwarder nodes is elected from each cube based on its proximity with respect to the destination to avoid void occurrence. Moreover, the data packets are recovered from void regions with the help of mobile sinks which also reduce the data traffic on intermediate nodes. Extensive simulations are performed to verify that our proposed work maximizes the network lifetime and packet delivery ratio.

## 1. Introduction

A group of interconnected sensor nodes through acoustic channel form a underwater wireless sensor network (UWSN). The collaborative behaviour of sensing devices in the network enables: monitoring of remote locations, physical environment, temperature, humidity, battlefield, oceans, volcanoes and many more [1,2], whereas sensors are the key component of UWSN, which are randomly deployed over the specified network volume, to monitor, sense, gather and transmit the information of interest. In UWSN, sensor nodes have limited battery, which is key consideration while designing a routing strategy. Also the sustainable deployment of sensor system is required to reduce the deployment and operational cost to prolong the network operational time [3,4].

In order to ensure successful communication among the nodes in acoustic network, the necessary factors are required to be considered in the design of a routing algorithm. For instance, the major factors associated with underwater channel need to be analyzed e.g., high delay of acoustic signal propagation because sound can propagate in acoustic environment with speed of 1500 m/s [4], high bit error rate because of noise and dynamic nature of acoustic medium, limited bandwidth, multi-path fading, etc. [5]. Therefore, an efficient routing strategy for acoustic channel is desired which balances energy dissipation to optimize the network lifespan [6]. For minimal energy consumption, geographic routing is widely accepted because of its scalable and simple implementation methodology [6,7]. Moreover, the stateless nature of geographic routing allows it to communicate without establishing entire path from source to destination. This algorithm only computes one eligible neighbor which acts as a potential forwarder to relay the data packet. Additionally, this routing mechanism is highly effective when node density is high because it follows greedy forwarding mechanism to transmit the data in multi-hop manner [5]. While in sparse deployment, due to the greedy approach, nodes select an optimal route in terms of distance which results in immutable selection of the same node resulting in sudden depletion of the node battery [8]. This death of the node creates energy hole which results in the breakage of the data route because of which downstream nodes cannot deliver their sensed information to the base station.

The aforementioned limitation is avoided through opportunistic routing (OR) paradigm, in which the selection of the forwarder set enures successful data delivery towards the destination node even if one node from the set fails, still, the data is delivered [9]. However, the delivery of redundant packets at base station degrade the performance of OR. To avoid the transmission of duplicate packets, control message exchange or holding time mechanism is used in opportunistic routing strategy. In the former approach, node with minimum distance and shorter route from the destination compared to nominated neighbors of the sender, is elected to deliver the data by acknowledging with control message that data is delivered successfully. In the later one, holding time is computed for each neighbor node to assign the priority in order to communicate on the acoustic channel. Incase of high priority node failure, node with second high priority in the set, transmits the data packet after its holding time expires. Still, in receiver based communication, duplicate packets from the hidden terminal regions are not suppressed. The hidden terminal is a region, where nodes lie in the transmission range of source node, but these nodes are unable to receive transmission or failure acknowledgement from the high priority node and ultimately transmit data packet towards the destination.

Due to duplicate transmissions from the hidden terminal volume, unnecessary energy dissipates resulting in short network lifespan. To mitigate the aforementioned constraint, a paradigm known as geo-opportunistic routing emerges, in which geographic routing is adopted for greedy forwarding by using geographic location of the set of forwarder nodes [9,10]. However, in multi-hop data delivery, nodes positioned nearby base station are overburden with traffic which dissipates the node energy very quickly. Due to the quick dissipation of node battery near the sink, nodes placed away from destination are unable to transmit data due to the unavailability of forwarders.

To reduce the data load at intermediate nodes and recover data from the void regions, mobile sinks are mounted over the ships, vehicle, etc. to gather the information of interest from the region of interest. The availability of mobile sinks enables new horizon of applications including but not limited to seabed survey, the detection of minerals from the oceans which are humanly not possible to monitor [11]. Hence, the mobility provided ease to directly retrieve the information from the communication void. With the incorporation of sink mobility, the network topology and delay in the network increases with the passage of time. To reduce the aforementioned constraints in geo-graphic, opportunistic, geo-opportunistic and mobility of sinks, we have made the following contributions:

**Contributions:** We have proposed two routing algorithms; geo-spatial division based geo-opportunistic routing scheme for interference avoidance (GDGOR-IA) and geographic routing for maximum coverage with sink mobility (GRMC-SM). The distinctive features of our work are list as follows:

- The distribution of the network field into small cubes is performed to make local routing decisions for efficient energy consumption.
- The distributive geo-opportunistic routing in geo-spatial network field avoids the interference by restricting number of nodes.
- In order to minimize traffic load on intermediate nodes, mobile sinks gather data directly from underwater nodes and also use to recover data from void hole.
- An optimal holding time is formulated to ensure that successful transmission acknowledgement receives before the time expires of an individual node.

This paper is organized as: a comprehensive overview of existing underwater routing schemes is stated in Section 2. While, Section 3 presents the pre-requisites of the network which are network model, energy model and control messages. Geo-opportunistic routing without sink mobility is discussed in Section 4 and Section 5 illustrates geo-opportunistic routing with sink mobility. In Section 6, a detailed linear programming based mathematical problem formulation subjected to attain optimal network lifetime and packet delivery ratio (PDR). Section 7 presents a detailed discussion of simulation results regarding network lifetime, PDR and data traffic load. Finally, Section 8 concludes our proposed work based on the analysis made in Section 7 with compared existing literature. The symbols and notations used in the manuscript are listed in Table 1.

## 2. Related Works

To understand the proposed methodologies, we have discussed existing state of the art which is relevant to our work in two subsections; geographic and opportunistic routing algorithms.

### 2.1. Geographic Routing

**Figure 1:**
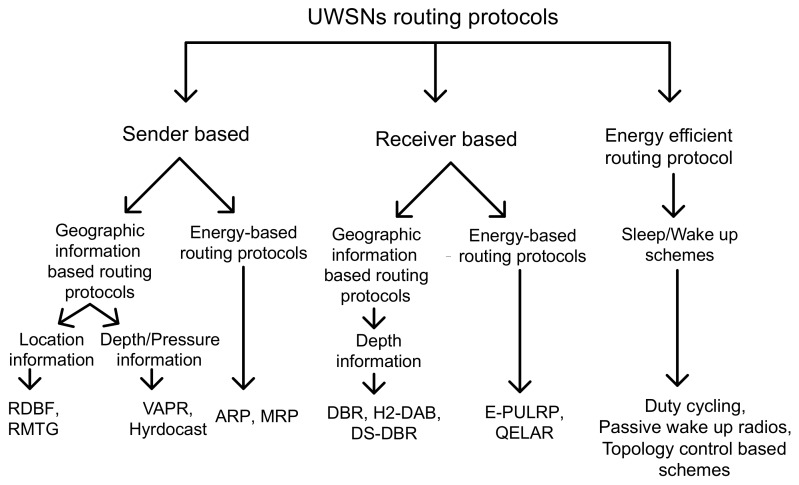
Classification of existing routing protocols.

Geographical routing utilizes location information for path establishment between source and destination. This scheme uses geographic position information to send packet towards the closer destination from each hop till packet reaches the sink. Unlike the proactive routing that bears large overhead to maintain full path, geographic paradigm relies on one or two hop information for routing data packets. This feature enhances scalability of large sensor networks. In geographic routing, services like geocasting can be used to get geographic information for data forwarding within a geographic region [12]. Considering geographic information, we categorize the existing protocols and schemes into two hierarchies: sender-based and receiver-based underwater routing protocols are tabulated in Table 2. These hierarchies are further divided into two categories based on information type: either location information or depth information as shown in Figure 1.

#### 2.1.1. Sender-Based Geographic Routing

Sender-based underwater routing protocols relying on geographic information for routing purpose are relative distance based forwarding (RDBF) [13], routing and multi-cast tree-based geo-casting (RMTG) [14], and adaptive routing protocol (ARP).

In RDBF, an efficient route search towards destination is performed using location information. For finding suitable node for forwarding process, based on distance, a fitness function is defined with respect to destination. Hence, nodes nearby sink have greater probability to get selected as forwarder nodes. In order to avoid redundant transmissions and collisions, a node maintains neighbor information and if the same packet is transmitted from other node, it simply drops the packet. Residual energy threshold is maintained for efficient energy consumption. However, accurate position information is required for each node for successful communication which is hard to obtain in underwater environment [13]. The RMTG geocast routing protocol relies on multiple piece of information, such as location information of nodes and their neighbors, route discovery for selection of node closest to the destination and route maintenance. This protocol has addressed problems like void hole and link breakage problems. A multicast shortest path is formed for packet transmission within the intended geographic region [14].

In ARP, data packets are assigned different delivery priorities that depend on application requirement. Higher priority packets are sensitive to delay. So, trade-off between throughput and latency in ARP exists. Also, this uses location information and it is an energy efficient protocol however, this incurs high overhead [15].

#### 2.1.2. Receiver-Based Geographic Routing Algorithms

Existing receiver-based underwater routing algorithms using geographic information for routing are; depth-based routing (DBR) [16], delay sensitive DBR (DSDBR), hop-by-hop dynamic addressing based (H2-DAB) [19], etc. In DBR, greedy forwarding is used to find out higher depth node. This approach leads to immutable nomination of forwarder resulting in energy hole creation. In DBR, depth information is used instead of location information, therefore, multiple nodes transmit the same packet that results in high energy depletion and collisions at receiver side [16]. The extended version of DBR was energy efficient DBR (EEDBR), in which energy was also considered for forwarder selection along with the depth to avoid immutable nomination of the forwarder node [17]. In [18], another variation of DBR that formulates holding time calculations to reduce latency in the network. H2-DAB [19] uses two part information: node ID and hop ID for routing the data packet. This protocol is energy efficient because it does not store complex routing information in routing tables. Anyhow, it is needed to update routing table on time for effective data transmission.

To avoid horizontal communication between same depth sensor nodes, DVPR opts triangular inequality theorem. According to that, same depth nodes are avoided using coordinate information of participating nodes in communication. However, accurate position information is a challenging task itself [20]. Considering this shortcoming, authors proposed self-adaption algorithm based on position information of sender node and receiver node with respect to virtual vector in a virtual pipeline. According to this information, suitableness of a node is calculated for routing the data towards destination [21].

Another objective aimed to achieve in our work is maximum coverage over the monitoring network region. We have performed multiple sink positioning in the way to attain our objective up to the maximal extent. Because sinks are mechanically driven devices and a specific cost is associated with them. Concerning to that, we have tried to minimize the total travelled distance of sinks deployed in three dimensional field. Such distance constrained problem is addressed in [22], in which sinks time profile is monitored. Additionally, sum of all sinks time profile is observed and based on that scheduling is made for the selected sink for sink location. In another contribution [23], authors performed formulation based on predetermined routing paths and the variable pause times for lifetime optimization.

In [5], the authors proposed depth adjustment techniques known as GEDAR for void node recovery in UWSNs. In this scheme, winch-based apparatus is used for depth adjustment of void node in vertical direction. Further, with the depth adjustment, the greedy forwarding approach is resumed. Additionally, when void node occurs, the fall recovery procedure is used to discover an alternate path for delivering data successfully. However, if alternate route is not available in that case, depth of the node is adjusted to resume the communication among the network nodes.

#### 2.1.3. Opportunistic Routing Protocols

Opportunistic protocols have been presented to avoid retransmissions in the network. In the existing literature, an EnOR: energy balancing routing protocol for UWSNs is proposed to avoid immutable selection of forwarder nodes by assigning priority to each node [8]. This routing algorithm considers three parameters for forwarder node selection; the link reliability, the energy, and packet advancement towards the destination. With the help of aforesaid parameters, the rotation of forwarder node selection is ensured. Additionally, EnOR extends the network lifespan and avoids the occurrence of void node by continuously rotating the forwarder node.

An OR protocol for void avoidance is proposed in [24]. In this routing protocol, instead of traditional void node recovery methods, depth adjustment mechanism is used to relocate node in vertical direction. This scheme constructs adjacency graph with neighbor nodes at each to elect potential forwarder nodes. The distinctive aspect of the scheme is to elect forwarders from the transmission range of every node which resolves the issue of hidden terminal problem. This scheme also has the ability to by pass any kinds of void by utilizing omni directional routes. However, the trade-off of high energy consumption persists in [24].

A delay sensitive OR protocol is presented [25] to achieve high goodput and minimum delay in the network. Authors consider ${EEL|}_{success}\left(Fi\right)$ (expected end-to-end latency [25]) parameter to ensure at least one of the forwarder delivers data packet successfully at the destination. To predict close value, two step heuristic scheme is presented based on the per node forwarding set determination and assigning priority to advancement node. This scheme shows high goodput and efficient-energy consumption in the network.

In [26], VAPR protocol exploits two hop depth information and hop counts to select next hop forwarder. It is easier to get depth information as compared to location coordinates. VAPR opts two fold procedure: improved beaconing and opportunistic forwarding. A node initiates a beacon containing information like its depth, direction and number of hops to initiate communication. Then, data is delivered solely based on next-hop direction, to deliver data in minimum number of hops. Due to efficient beaconing, VAPR is robust against failures and node mobility. In [27], hydrocast uses pressure information of sender and neighbor nodes along with two hop neighboring distance. During forwarding process, hydrocast selects a set of neighboring nodes based on greedy advancement towards destination, considering hidden terminal problem as well. Both VAPR and hydrocast maintain routing path to avoid void holes and the trade-off is high energy consumption.

Khasawneh et al. proposed reliable and energy-efficient pressure-based routing (RE-PBR) [28] algorithm by taking into account; link quality, depth, and residual energy for the selection of immediate forwarder node towards the destination. The inclusion of link metric balanced the battery depletion because of continuous rotation of forwarder node. This scheme achieved balanced energy consumption due to the avoidance of immutable forwarder selection.

Authors proposed autonomous underwater vehicles (AUVs) for data gathering from underwater sensor nodes to minimize transmission power of node [29]. In this scheme a cluster is formed and data collection from an individual cluster head node is performed for balanced energy dissipation across the network nodes. The validity of the work is confirmed through Monte Carlo simulations and deduced that energy is efficiently utilized directly effecting the network lifespan. However, the delay is high because no multi-hop mechanism is incorporated if AUV is not within the communication range. Additionally, the immutable selection of cluster head node will create void holes and degrades the network performance.

## 3. Preliminaries

### 3.1. Network Architecture

**Figure 2:**
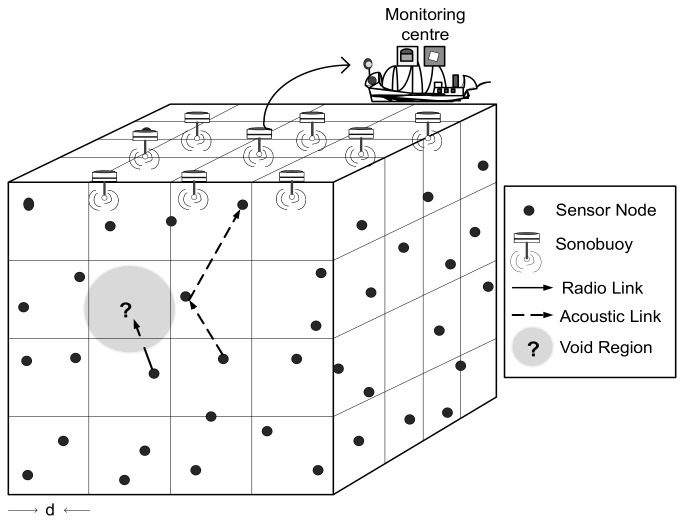
Network architecture.

All nodes are deployed randomly in three dimensional network field also shown through Figure 2. The volume of the network is divided logically into small cubes to perform distributive routing in each cube. The number of cubes are represented as $C_{n}=\left\{c_{1},c_{2},c_{3},\ldots ,c_{n}\right\}$ where each cube nodes are connected to its adjacent neighbor cube nodes through acoustic link. The sensor nodes operate in two modes: the first one is, sensing mode where node predicts the environmental effects and the second one is transmit mode in which sensed data is delivered to the destination through acoustic data link. The network is homogeneous and consists of $N_{n}=\left\{N_{1},N_{2},N_{3},\ldots ,N_{n}\right\}$ nodes deployed inside the water along with set of sonobuoys $S_{s}=\left\{s_{1},s_{2},s_{3},\ldots ,s_{n}\right\}$ which are positioned at the water surface. Also, an assumption is made that each node is capable to transmit data successfully within its communication range. All the sensing devices are provided with limited memory, modem for acoustic communication, transceiver and battery. While sonobuoys have both acoustic and radio modems. The former is used to retrieve information of interest from the underwater sensors, and latter to deliver data for further processing to the offshore data center. Further, an assumption is made that every node knows its location in advance. Whereas, the depth of void node also adjusted through the same mechanism as discussed in [30,31]. The cost associated with depth adjustment is similar as provided in [32]. For simulations, we consider the unit disk graph model in which data packet always received successfully within the transmission range [33].

### 3.2. Beacon Message Types

Beacon message contains identifiers to establish connections among the network nodes. Each node uses beacon identifiers for performing transmission of data packet, sensing along with reception of data packet [7]. From the beacon identifiers, each node maintains a neighbor table which consists of cube number, unique ID and *X*, *Y*, *Z* coordinates. The ultimate objective to broadcast beacon messages among the network nodes is to acquire information of neighbor nodes and closest sonobuoy [6]. To acquire coordinates of each node in the acoustic environment, we have used the same mechanism as discussed in [34]. The periodic broadcast of the beacon message increase the overhead resulting in low network performance. Therefore, we only transmit the changed identifier in the beacon message to keep neighbor table fresh and avoid the data packet loss. Due to the transmission of updated identifier, unnecessary flooding of broadcast message is avoided and the purpose of neighbor information also fulfilled. Similarly, any sonobuoy belonging to the set $S_{s}$ has quintuple of information that includes ID of the sonobuoy, *X*, *Y*, *Z* coordinates, sequence number of the beacon message and Λ as a flag to indicate that latest neighbor information is propagated among the neighbor nodes. With the help of neighbor information, every node transmits its sensed data to reach its nearest sonobuoy through its neighbor nodes.

### 3.3. Potential Neighbor Set Selection

After the dissemination of beacon message, every node has its neighbor table. However, still it is required to nominate potential forwarder node because every neighbor is not the potential forwarder for relaying data packets. The potential neighbor is defined as the node which has shorter route than the source node. In our both proposed schemes, we adopt the greedy approach to transmit data towards the destination node. Neighbor nodes which satisfies the criteria of greedy forwarding are computed using Equation (1). The ultimate goal of greedy forwarding is to advance the data packet through shortest and energy optimal path to reach the destination.

(1) $$F_{set}\left(k\right)=n_{i}\epsilon N_{k}\left(t\right):\exists S_{n}\epsilon S_{s}\left(t\right)|D\left(n_{i},s_{n^{*}}\right)-D\left(n_{k},s_{n}\right)>0$$

The potential neighbor set selection follows $n_{k}\left(t\right)$ steps to include $N_{k}\left(t\right)$ and $S_{k}\left(t\right)$ neighbors and sonobuoys at time *t* in the neighbor table [35]. In Equation (1), $F_{set}\left(k\right)$ provides potential neighbor set of a source node k.

### 3.4. Geospatial Division Model

As discussed earlier, in proposed schemes network filed is logically divided into $C_{n}$ cubes through geospatial division method. The following relationships between two cubes are:

- Two cubes are adjacent to each other at common vertex, that is vertex adjacent.
- Two cubes are neighbor with common one edge, that is edge adjacent.
- If two cubes have adjacent surface to one another, that is surface adjacent.
- Otherwise, cubes are completely disjoint.

**Figure 3:**
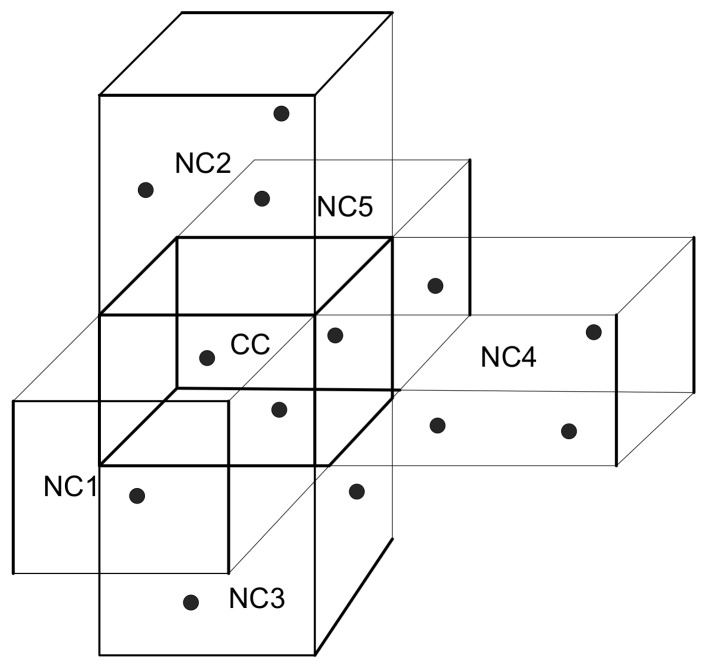
Cubical representation of target cube.

The first three; cubes have adjacent vertex, edges and surfaces. Moreover, each cube has 8 adjacent neighbor vertex, 12 edges and 6 surfaces. Figure 3 denotes a current cube (CC) with its neighbor cubes (NCs), NC1, NC2 up to NC5. The selection of cubes is discussed in Section 4.

## 4. GDGOR-IA

In this section, we discuss the selection of the target cube in detail as follows.

### 4.1. Target Cube Selection

GDGOR-IA works in two phases: in phase I, the Algorithm 1 runs for the selection of target cube. For that purpose, a source node laying in the CC acquires its coordinates and source cube ID. A set of NCs of CC calculates Euclidean distance with respect to their nearby sonobouys. After the computation of Euclidean distance from CC, every neighbor node from NCs calculates its physical distance to satisfy the greedy forwarding criteria to become the potential forwarder node to relay the data packet. NC with smallest Euclidean distance is selected as target cube (TC) for the CC. All the cubes are priorities based on the computed distance, which are used as backup to transmit data incase of high priority neighbor cube failure. This is the where actually opportunistic routing really helps to find out an alternate route to proceed with the greedy data forwarding. It is to be noted that whenever Euclidian distance is measured with the sonobuoy, the distance is measured from the centre of the cube. In case, two NCs are meeting the selection criterion, choose any one of them randomly.
**Algorithm 1:** Target cube selection.
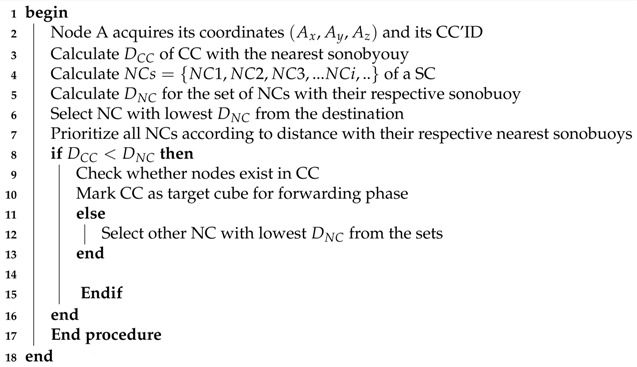


### 4.2. Next-Hop Forwarder Set Selection Criterion

In geographic routing single forwarder node is nominated to transmit data towards the destination. The primary disadvantage associated with the single forwarder selection is packet loss in case of bad link quality or void hole. Therefore, we have incorporated the geo-opportunistic routing paradigm to utilize the broadcast nature of wireless channel to nominate multiple forwarder node. This forwarding enables the selection of the potential forwarder set to ensure the reliable data delivery with minimal retransmissions in worst scenarios. However, it incurs more delay because all neighbor nodes wait till packet reaches the farthest node. To overcome this problem, we intend to select TC with less number of nodes but within a threshold set after considering link quality in Equation (2). This shows the error probability $P_{BER}$ and collision rate probability $P_{CR}$ where *L* is the size of packet [36]. The selection of TC with minimum number of neighbors helps in reduction of interference because minimum number of neighbors access the wireless channel. Moreover, the delay is reduced up to significant amount due to the participation of few nodes from the NC. Furthermore, within the TC, election of next-hop forwarder set is done through advancement towards the destination (ADV). The ADV is calculated for the set of nodes $N_{k}=\left\{N_{1},N_{2},N_{3},\ldots \right\}$ in the TC. The nodes are prioritized on the basis of highest advancement towards the destination.

(2) $$\alpha =\frac{1}{P_{CR}}\times {\left(1-P_{BER}\right)}^{L}$$

(3) $$ADV\left(n_{i}\right)=D\left(n_{k},s_{n}^{*}\right)-D\left(n_{i},s_{i}^{*}\right)$$

$ADV(n_{i})$ shows the advancement of $n_{i}$, and neighbor of the source node is represented with $n_{k}$ towards its closest sonobuoy in Equation (3). For node $n_{i}$ belonging to the potential neighbor set $F_{set}\left(k\right)$ taken from Equation (1), normalized advancement towards the destination is calculated according to Equation (4) [5].

(4) $$NADV\left(n_{i}\right)=ADV\left(n_{i}\right)\times P\left(d_{k}^{i},L\right)$$

Algorithm 2 illustrates the selection of next hop forwarder in GDGOR-IA. Firstly, source node acquires the information about the neighbor nodes which is performed as discussed in Algorithm 1. Once, we have the neighbor information, source node proceeds to the next step for the nomination of potential forwarder node to execute the network operations. Let’s assume that source node $n_{a}$ deployed in downstream target cube which has neighbor cubes consists of numerous set of neighbor nodes $PF_{set}\left(n_{a}\right)$ named as potential forwarders of $n_{a}$. This set is a subset of $F_{set}\left(n_{a}\right)$ in all nodes meet the selection conditions imposed through Equation (2). If $PF_{set}\left(n_{a}\right)$ is an empty set, we take help from the information of Algorithm 1 providing the set of available NCs which can be used as target cubes. Each node differentiates itself from the other based on the cube ID. In case of multiple available target cubes, we obtain multiple forwarder sets $F_{set}\left(n_{a}\right)$ for $n_{a}$. In such conditions, we compare the accumulated NADV of all sets to select the cubes which has less node number for avoiding the interference and minimizing the delay.
**Algorithm 2:** Next-hop forwarder selection.
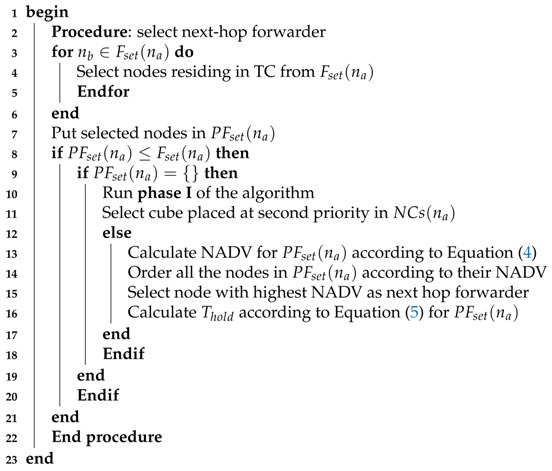


As the final step, the nodes in the set are ordered according to their NADV. Next hop forwarder node is selected based on highest normalized advancement and rest of the nodes are prioritized accordingly. The next hop forwarder node holding time is calculated using Equation (5).

(5) $$T_{h}^{i}=T_{p}+\sum_{j=1}^{i}\frac{D(n_{j},n_{j+1)}}{s}+i\times T_{proc}.$$

$T_{p}$ depicts the propagation delay incase of one hop away sender from the destination. The second part of the expressions contains the propagation delay of all the member nodes where *s* is the speed of sound in the acoustic medium. The third expression $T_{proc}$ depicts the processing time of each node *i* at each hop.

All nodes belonging to the same cube can overhear each others transmission that handles the hidden terminal problem effectively. All other nodes gather packets from neighbor nodes to acquire information about cube ID. This process caters problem of hidden terminal along with the interference among potential neighbor nodes residing in the same cube, thus the packet loss is reduced.

## 5. GRMC-SM

**Figure 4:**
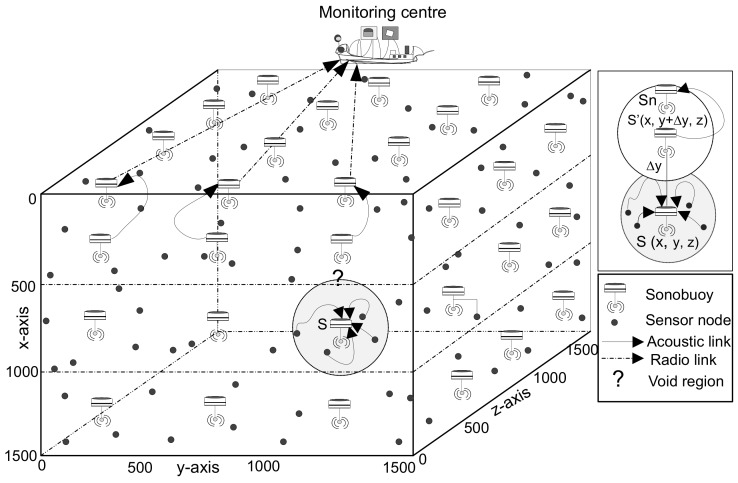
Schematic diagram of GRMC-SM.

We deploy $MS_{n}$ number of mobile sinks $MS_{n}$ = $\left\{ms_{1},ms_{2},\ldots ,ms_{n}\right\}$ to retrieve information directly from nodes. Figure 2 illustrates multi-sink architecture which is also discussed in Section 3.1, $S_{n}$ sinks are replaced with mobile sinks $MS_{n}$. The updated network model is depicted is Figure 4. As illustrated in Figure 4, all sinks are deployed uniformly within the network region, where nodes communicate with the nodes of neighbor cube in their transmission range to handover the data packet to the closest $MS_{n}$. In case of coverage hole, sinks change their coordinates in order to gather data packet from the node directly. The sink movement is governed with the intent to minimize the total travelled distance which directly minimize the delay. Though, there is a particular cost associated with the mechanical movement of sinks but mobile sinks come to the water surface to deliver data and also get recharged, thus, sinks have no constraint of energy to perform network operations.

### 5.1. Data Forwarding and Routing in GRMC-SM

In GRMC-SM, all nodes forward their packets to one-hop neighbors or in-range sinks placed at shorter the distance from the surface than the node itself. The deployment of mobile sinks is uniform in the field to cover maximum volume of the network. If, a node is unable to find sink(s) in its transmission range then nodes relay data packet via multi-hop mechanism towards the destination by following the greedy approach. Algorithm 3 presents the data forwarding (DFM) and routing mechanisms in GRMC-SM.

In case, a node is trapped in a coverage hole and does not find a potential neighbor node or nearby sink. This node broadcasts a void-node-declaration message to its neighbors in the CC and to the NCs to avoid the data loss and transmission trap. This declaration saves node battery and allows the network nodes to operate for maximal time period. This information is further spread to the nearby mobile sink, which aid the void node to deliver its sensed and received information to the base station for further processing. Once $MS_{n}$ receives the void-declaration message, the movement of the closest mobile sink is triggered to change its course to provide to the void node at top priority. When mobile sink $S^{\prime }\left(x,\Delta y,z\right)$ disseminates the changed co-ordinates in its transmission range, the void node forwards its data to *S*’. From there onward, mobile sink relays composite data to the sinks placed at the surface. As a last step, a set of surface sinks transmits data via radio link to monitoring centre on the surface.
**Algorithm 3:** Data forwarding mechanism (DFM).
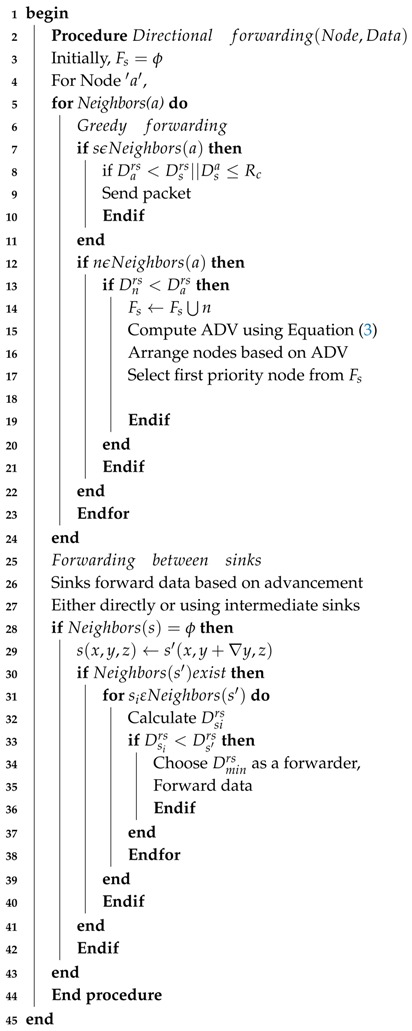


### 5.2. Recovery Mode via Sink Mobility

Several methods of void hole recovery have been proposed e.g., physically replacing the dead nodes or recharging the sensor node battery; mechanical movement of the sensor nodes to adjust the depth [5] and usage of relay nodes to perform particular function of relaying data in case of void occurrence.

We have incorporated the sink mobility in GDGOR-IA scheme to analyze the effect of controlled sink mobility when void hole occurs. During the operation of forwarding, when a node traps in the void region and finds no alternate route to proceed the network communication even after examining its neighbor information. To resume the greedy forwarding, void node recovery mechanism operates. To inform the low depth neighbors, void-node-declaration packet is disseminated to inform the mobile sink. If neighbor node receives this declaration message and is not a void node itself, it replies the void-node-declaration-reply message with its location and neighbor information. This step is basically a message-based recovery for sender void node.

In other case, if the downstream node is also in the void node, then scenario leads towards local maxima trap with couple of void nodes in it. Thus, all data packets will be dropped because potential forwarders are not available to relay the transmitted data packet. To overcome earlier said scenario, uniform mobile sink deployment is performed in GDGOR-IA scheme and evaluated the performance of the proposed GDGOR-SM. Deployment of sinks in three dimensional network field is intended to reduce and recover data from the void regions. In mountain like trapped region, nodes look for nearby sink using two hop information. When a sink receives void-node-declaration message disseminated by node having coordinates (*X*, *Y*, *Z*), it calculates its new depth based on location information of the void node. In worst scenarios, depth adjustment of sink node is not progressive towards the destination. However, data discarded due to communication void is forwarded to the sink.

## 6. Mathematical Formulation Using Linear Programming

Linear programming is a common mathematical strategy which gives an optimal outcome for a linear problem. Here, we have discussed how linear programming helps in optimizing throughput and balancing energy consumption.

### 6.1. Energy Consumption Minimization

The imbalanced energy depletion among the network nodes degrade the network performance. In this regard, various routing algorithms are proposed to address this problem. Thus, energy minimization is performed based on objective function by following linear constraints. In both proposed schemes, energy consumption caused during transmission and reception of data packet. We formulate the objective function to optimize energy consumption (Equation (6)).

(6) $$Min\sum_{i=1}^{N}E_{consumed}\left(i\right)\;\;\forall \;i\;\epsilon \;N$$

where $E_{consumed}$ is the energy consumed per packet per node in the network.

Initially, the energy depletion is because of packets transmission and reception which is counted as shown in Equation (7).

(7) $$E_{consumed}\left(ij\right)=\sum_{i=1}^{N}\left(E_{TX}+E_{RX}\right)$$

In Equation (7), $E_{consumed}$ between node *i* and node *j* is mainly due to the transmission of data $E_{TX}$ over the distance $\left(D_{\left(ij\right)}\right)$. The receiving energy $\left(E_{RX}\right)$ depends on number of bits received in the data packet from sender node according to Equation (8).

(8) $$E_{TX}^{max}=P_{TX}\times \left(HS+L\right)/DR$$

(9) $$E_{RX}^{max}=P_{RX}\times \left(HS+L\right)/DR$$

Equations (8) and (9) show optimal values of $E_{TX}$ and $E_{RX}$ and depend on transmission $P_{TX}$ and receiving $P_{RX}$ powers, respectively. Whereas, packet size is (*HS + L*) and DR depicts data rate.

(10) $$E_{total}=E_{initial}\times N$$

$E_{total}$ depicts the summation of network nodes energy as initial energy $\left(E_{initial}\right)$ given in Equation (10). The $E_{consumed}$ in each round (*r*) is stated in Equation (11) as,

(11) $$E_{consumed}=\sum_{r=1}^{r_{max}}\left(E_{consumed}\left(r\right)\right).$$

For GDGOR-IA scheme, energy consumption due to depth adjustment of void nodes shown in Equation (12),

(12) $$E_{consumed}^{\prime }=\sum_{r=1}^{r_{max}}\left(E_{consumed}\left(r\right)+E_{DA}\left(r\right)\right),$$

where $E_{DA}\left(r\right)$ depicts the amount of energy dissipated in depth adjustment during each round which is added till maximum round $r_{max}$ reaches.

(13) $$E_{DA}=N_{vn}\times \left(E_{DA}\left(n_{vn}\right)\right).$$

$N_{vn}$ represents the number of void node.

Objective function in Equation (6) is defined under following linear constraints:

(14) $$E_{\left(TX,RX\right)}\leq {E_{i}}^{initial}\;\;\forall \;i\;\epsilon \;N$$

(15) $$D_{\left(s,d\right)}\leq R_{c}\;\;\forall \;i\;\epsilon \;N$$

$D_{s,d}$ represents the distance between nodes *s* and *d* which must be less or equal to the $R_{c}$ communication range.

(16) $$E_{DA}\left(n_{vn}\right)\leq E_{i}^{r}\;\;\forall \;i\;\epsilon \;N$$

In GRMC-SM, $E_{consumed}$ is mainly due to single or multi-hop communication in the network. Therefore, $E_{consumed}$ associated with this scheme can be computed by Equation (11).

(17) $$E_{\left(TX,RX\right)}\leq {E_{i}}^{initial}\;\;\forall \;i\;\epsilon \;N$$

The summation of transmission and reception energies $E_{\left(TX,RX\right)}$ should remain less for successful transmission. While, $E_{\left(TX,RX\right)}$ restricts receiving energy through Equation (18).

(18) $$E_{\left(TX,RX\right)}\leq {E_{i}}^{r}\;\;\forall \;i\;\epsilon \;N$$

To limit the communication of the transmitter node within the transmission vicinity $R_{TX}^{max}$, Equation (19) is used. Moreover, the distance should be greater than zero as given $R_{TX}^{min}$ in Equation (20),

(19) $$D_{i}^{j}\leq R_{TX}^{max}\;\;\forall \;i,j\;\epsilon \;N$$

(20) $$D_{i}^{j}\geq R_{TX}^{min}\;\;\forall \;i,j\;\epsilon \;N.$$

(21) $$E_{TX}^{min}=E_{TX}/Ls$$

(22) $$E_{RX}^{min}=E_{RX}/Ls$$

**Graphical Analysis**: Let consider a scenario where 250 m be the transmission range and $L_{s}$ levels i.e., $L_{s}$ = [1–5]. The intention to make transmission $L_{s}$ is to note down the pattern of energy dissipation based on $L_{s}$ expressed in Equations (21) and (22). Where (HS+L) = 888 bits, DR = 50 kbps, *N* = 450, $P_{TX}=2$ W, and $P_{RX}=0.0158$ W. Based on earlier given parameters, $E_{TX}$ is 4.555 J computed via Equation (21) at $L_{s}$ = 1 and 1.5 J via Equation (21) when $L_{s}$ = 5. By Equation (22), $E_{RX}$ is 0.24 J computed at $L_{s}$ = 1 and 0.7 J computed when $L_{s}$ = 5.

(23) $$1.74\leq E_{TX}+E_{RX}\leq 5.25$$

(24) $$0.24\leq E_{RX}\leq 0.7$$

(25) $$1.5\leq E_{TX}\leq 4.555$$

**Figure 5:**
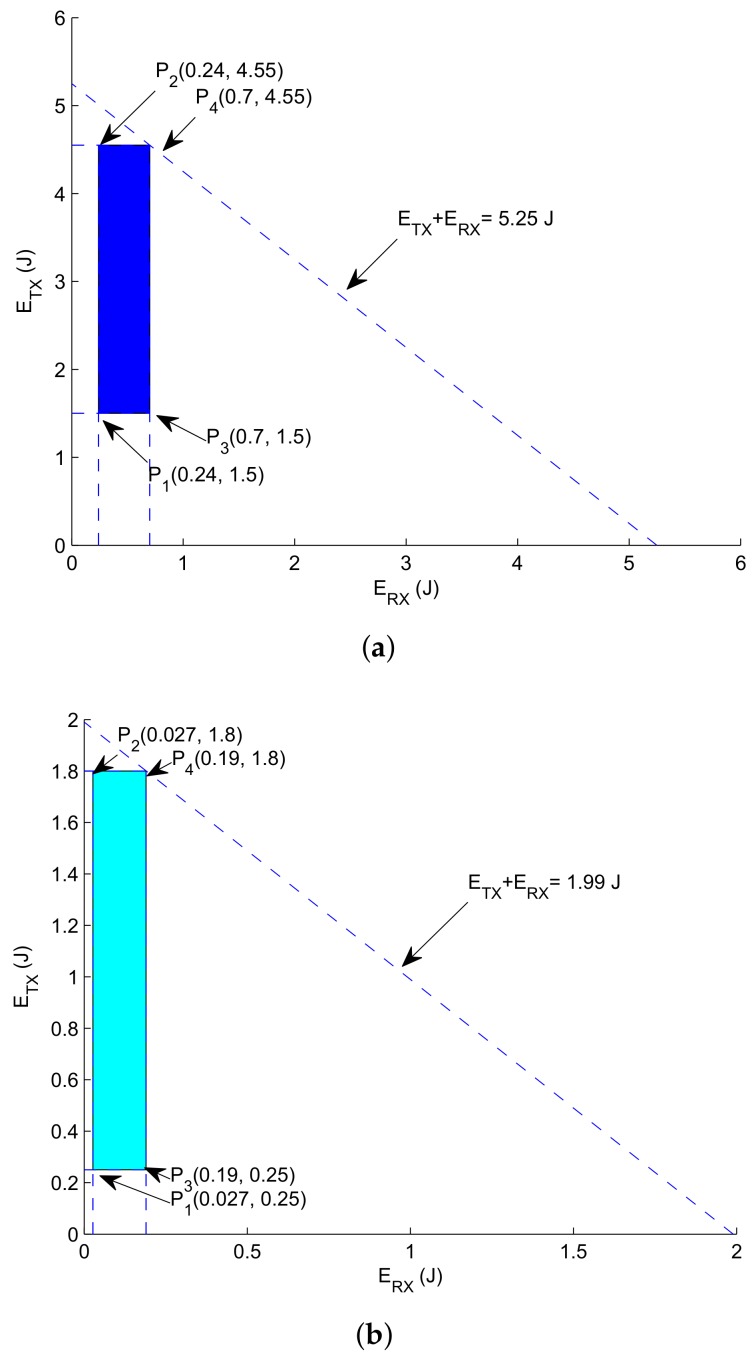
Feasible regions. (**a**) Feasible region for energy tax minimization (GDGOR-IA); (**b**) feasible region for energy tax minimization (GRSM-MC).

Figure 5 depicts the feasible region in which energy consumption always results in optimal network lifespan. Thus, points from given region yield minimal energy consumption with valid solution. 

The solution is tested on the following vertex which are computed in Figure 5a.

at $P_{1}:0.24+1.5=1.74$ J
at $P_{2}:0.24+4.555=4.79$ J
at $P_{3}:0.7+1.5=2.2$ J
at $P_{4}:0.7+4.555=5.25$ J

For GRMC-SM, following vertex are used which are depicted in Figure 5b.

at $P_{1}:0.027+0.25=0.27$ J
at $P_{2}:0.027+1.8=1.827$ J
at $P_{3}:0.19+0.25=0.44$ J
at $P_{4}:0.19+1.8=1.99$ J

Hence, the energy consumption within the bounded region is minimal resulting in optimal network lifespan, which is further verified through simulations in Section 7.

### 6.2. PDR Maximization

In order to enhance network throughput by consuming minimum energy, packets are transmitted through multiple hops. Throughput is number of packets successfully reached the sink. Link quality is taken into consideration by defining threshold value δ for selecting optimal neighbor nodes at each hop. Additionally, it ensures successful packet delivery. Moreover, energy needed to transmit the packet must be fulfilled during forwarding according to C1. All aforesaid constraints are considered during the formulation of the objective function given in Equation (26).

(26) $$Max\sum_{i=1}^{N}T_{p}\left(i\right);\;\;\forall \;i\;\epsilon \;N,$$

where $T_{p}\left(i\right)$ is network throughput, $T_{p}\left(r\right)$ represents the amount of packets received during *r* rounds which is expressed mathematically in Equation (27).

(27) $$Max\sum_{r=1}^{r_{max}}T_{p}\times P;\;\;\forall \;i\;\epsilon \;N,$$

such that:

C1: $E_{TX,RX}\leq E_{r}$
C2: $P_{link}\geq \delta$
C3: $E_{TX,RX}\geq E_{th}$

where $E_{th}$ is the threshold for transmission and reception energies.

C4: $0<D_{ij}\leq D_{ij}^{max}$

**Figure 6:**
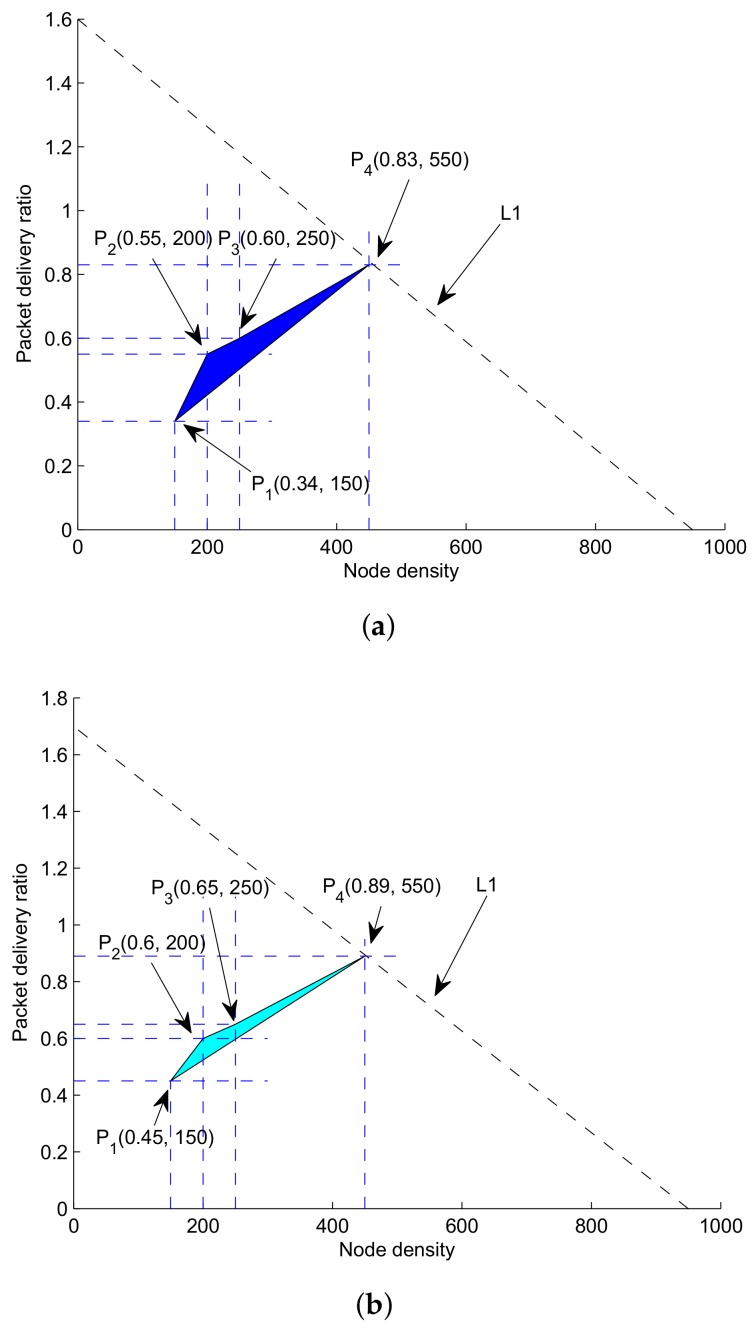
Feasible regions. (**a**) Feasible region for throughput maximization (GDGOR-IA); (**b**) feasible region for throughput maximization (GRMC-SM).

C1, C2, C3 and C4. C1 and C3 restrictions on $E_{TX}$ and $E_{RX}$ are set to avoid unnecessary energy consumption. In GRMC-SM, all nodes report their sensed data to the nearest sink. PDR of the network is accumulated packets successfully received at all the sinks. Equation (27) shows the summation of all the data packets in *r* rounds. Feasible region for GDGOR-IA lies within these following vertex points as shown in Figure 6a.

at $P_{1}\left(0.34,150\right)$
at $P_{2}\left(0.55,200\right)$
at $P_{3}\left(0.60,250\right)$
at $P_{4}\left(0.83,550\right)$

Similarly, for GRMC-SM, feasible region lies within following vertex points illustrated in Figure 6b:

at $P_{1}\left(0.45,150\right)$
at $P_{2}\left(0.6,200\right)$
at $P_{3}\left(0.65,250\right)$
at $P_{4}\left(0.89,550\right)$

### 6.3. Minimization of Average Delay

During the operation of forwarding in the network, sender nodes transmit packets directly or via multi-hops. We define average delay incurred due to direct and multi-hop transmission in *r* rounds for *N* number of nodes in the network as in Equation (28). In multi-hop transmission, node waits for $T_{w}$ time as shown in Equation (29),

(28) $$Min\sum_{i=1}^{N}D_{tot}\left(i\right)\;\;\forall \;i\;\epsilon \;N.$$

(29) $$T_{w}=D_{Proc}+D_{Prop}+T_{hold},$$

(30) $$T_{Prop}=\left(R_{c}-D\left(ij\right)\right)/s,$$

(31) $$T_{hold}=\sum_{i=1}^{j}D\left(n_{i},n_{i+1}\right)/s.$$

Total delay incurred comprises of delay due to direct transmission and multi-hop transmission as in Equation (32),

(32) $$D_{tot}=D_{DT}+D_{MHT}$$

For direct transmission to in range sinks, time taken is accumulation of propagation time and processing time. Therefore, delay caused due to that is $T_{w}^{\prime }$ as in Equation (33):

(33) $$T_{w}^{\prime }=D_{Proc}+D_{Prop}$$

(34) $$D_{DT-min}=T_{w}^{\prime }\times H_{n};$$

where $H_{n}=1$ for direct transmission scenario when the sink is in transmission range of source node.

(35) $$D_{MHT-min}=H_{n-min}\times T_{w};$$

(36) $$D_{MHT-max}=H_{n-max}\times T_{w};$$

The objective function in Equation (28) is formulated under following constraints C1, C2, C3:

at C1: $0<D_{max}^{ij}\leq R_{c}$
at C2: $0<T_{w}$
at C3: $H_{n-min}\leq H_{n-max}$

**Figure 7:**
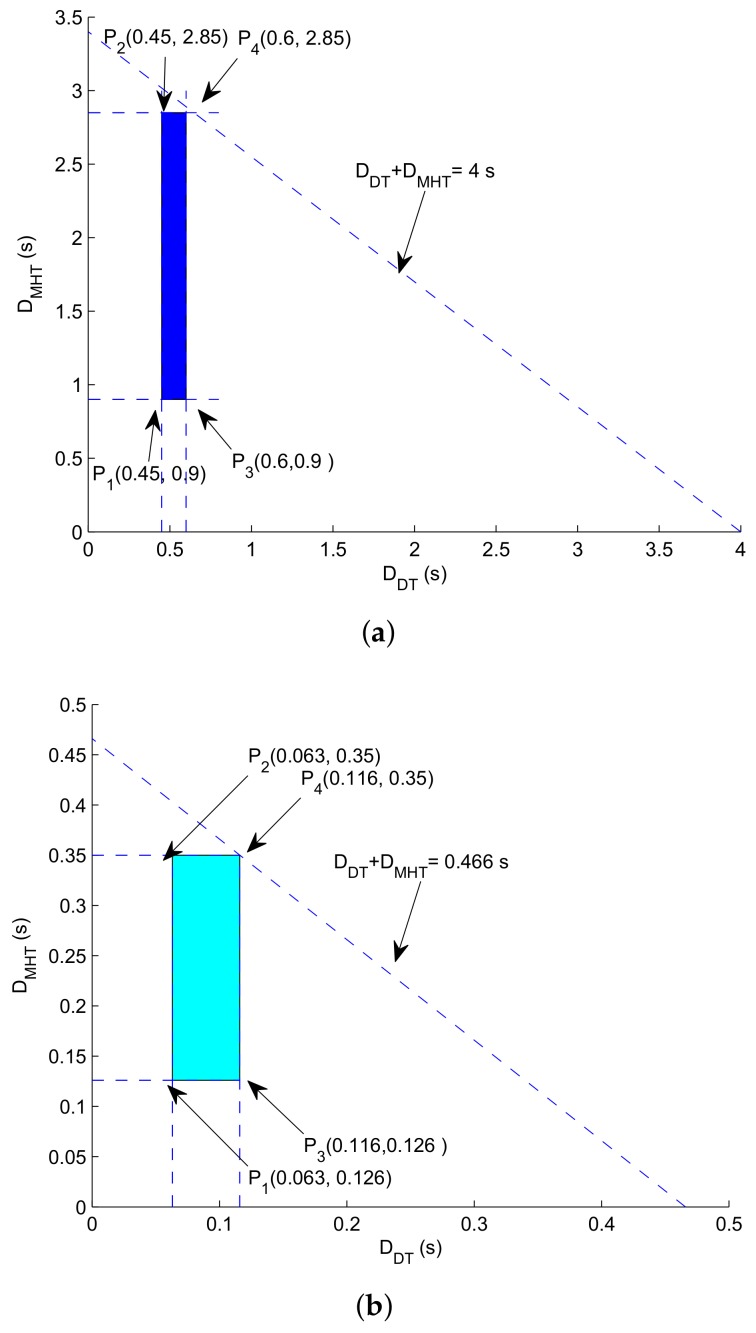
Feasible regions. (**a**) End to end delay: feasible region for GDGOR-IA; (**b**) end to end delay: feasible region for GRMC-SM.

**Graphical analysis**: Let’s consider, if source node be in the transmission range of sink and it relays data directly. During this, delay caused is represented via $D_{DT}$. On the other hand, when sink cannot be accessed directly by the sender node, then packet is transmitted through multiple hops. By assuming that minimum delay is caused on one-hop transmission and maximum delay occurs when data is delivered through multiple hops. We can compute maximum and minimum delays caused in both direct transmission scenario and multi-hop scenario; as, shown in Figure 7.

$1.35\leq D_{DT}+D_{MHT}\leq 3.45$
$0.45\leq D_{DT}\leq 0.6$
$0.9\leq D_{MHT}\leq 2.85$

Each vertex of the region is shown as:

at P1:0.45+0.9=1.35 s
at P2:0.45+2.85=3.30 s
at P3:0.6+0.9=1.5 s
at P4:0.6+0.285=0.885 s

Each vertex of the region is shown as:

$1.72\leq D_{DT}+D_{MHT}\leq 3.71$
$0.50\leq D_{DT}\leq 0.65$
$1.22\leq D_{MHT}\leq 3.06$

Each vertex of the region is shown as:

at P1:0.5+1.22=1.72 s
at P2:0.5+3.06=3.56 s
at P3:0.65+1.22=1.87 s
at P4:0.65+3.06=3.71 s

## 7. Simulation Results and Discussion

Simulation results of proposed work after carrying out extensive simulations in Aqua-Sim [37] are presented against three existing state of the art schemes; GEADR [5], EnOR [8], RE-PBR [28] and AUV-CH [29]. The performance is evaluated based on PDR, fraction of local maximum nodes, energy consumption per packet per node, end-to-end delay and depth adjustment. Further the analysis of proposed methodologies is done by varying traffic load as well. The detailed discussion is presented as follows.

### 7.1. Simulation Settings

To perform simulations, nodes are varied from 150–450 with 45 sonobuoys positioned at the water surface to gather data from underwater nodes. The network dimensions are 1500 m × 1500 m × 1500 m. Moreover, the transmission range is $R_{c}$ = 250 m and DR = 50 kbps. Also, we consider a payload of 150 bytes in each data packet including 20 bytes of beacon message. The energy dissipation associated with transmission, reception, idle state and depth adjustment is $P_{t}=2$ W, $P_{r}=0.1$ W, $P_{i}=10$ mW and $E_{m}=1500$ mJ/m, respectively [5]. The average of 50 distinctive simulation runs is taken for getting near optimal results against each value plotted in the graphs.

#### Performance Metrics

In this section, basic performance parameters are defined as:

- PDR: The ratio of packets successfully received at surface sonobuoys over amount of packets transmitted from each network node during the network operational time. The mathematical expression is given as follows:
  (37) $$PDR=\frac{P_{sonobuoys}}{P_{total_{gen}}},$$
  where, $P_{sonobuoys}$ shows the quantity of packets delivered at the destination, while $P_{total_{g}en}$ depicts the summation of packets generated from each network node.
- Fraction of void nodes: It is the amount of network nodes fail to deliver the data packet over the acoustic communication channel because of unavailability of further forwarder nodes in there transmission range.
- Energy consumption: It is defined as, the energy utilized in transmitting a data packet by a node within its transmission range. It is measured in joules (J).
- End-to-end delay: Time required for transmitting and propagating data from source to destination is called end-to-end delay and its unit is seconds (s).
- Depth adjustment: Net distance covered by a void node to find a forwarder node for resuming network operations is called depth adjustment and it is measured in meters (m).

### 7.2. Analysis of Proposed Scheme Results against Existing State of the Art

The simulation results of proposed schemes; GDGOR-IA, GRMC-SM, and GDGOR-SM against existing methodologies GEDAR, AUV-CH, and EnOR are presented in this section. The discussion is divided into different subsections; fraction of void nodes, depth adjustment, PDR, energy consumption, and end-to-end delay.

#### 7.2.1. Fraction of Void Nodes

**Figure 8:**
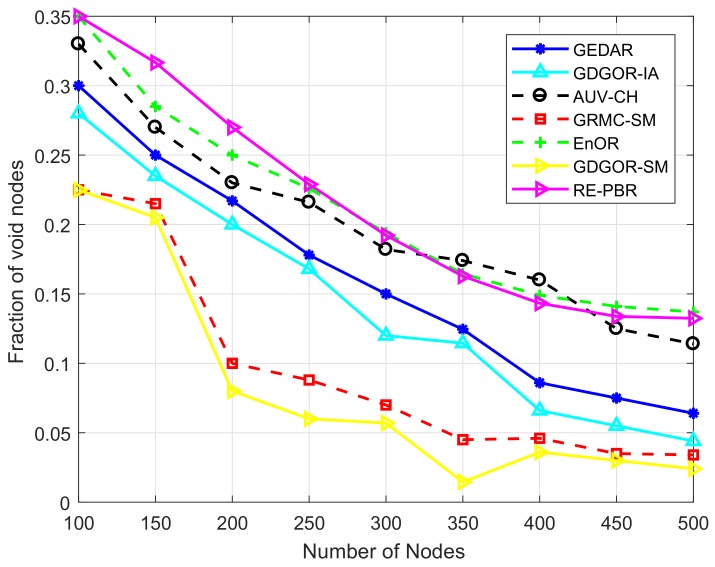
Fraction of void nodes plots.

Figure 8 depicts the fraction of failure in proposed and baseline schemes. The behaviour of GDGOR-SM shows that when node density is varied from 100–150, the fraction of node failure is decreasing gradually, however, as the quantity of nodes increased to 200, then sudden down fall is observed in the results of Figure 8. Further, after deploying more number of nodes up to 200–500, the trend shows continuous decrease. This scheme has less failure because of mobile sonobuoys which dive into the water from the surface to retrieve data directly and return data to specified destination. Similarly, in GRMC-SM, the trends of decreasing node failure at various node densities are almost similar to GDGOR-SM, however, the failure rate is higher due to the consideration of multihop transmission when mobile sonobuoy is not in range of a node.

Whereas, AUV-CH and EnOR performance starts declining because in opportunistic routing multiple sensor nodes participate in communication, and the reliability of delivering data is although high but the chances of communication failure are also high in both schemes. On the other hand, EnOR is focusing on rotating the forwarder node and has no mechanism for void avoidance, therefore it has high fraction of void nodes. The GEDAR utilizes sonobuoys which are positioned at the surface of water, whereas, lack of sonobuoys mobility exposes GEDAR scheme to communication failure. Thus, it is evident that 30% nodes lie in the category of void nodes in sparse network in both GEDAR and GDGOR-IA. Thus, the fraction of node failure is high when less number of nodes are deployed in the network and after increasing the number of nodes, it tends to reduce significantly in all the schemes. Fraction of void nodes is reduced in GEDAR and GDGOR-IA by opting depth adjustment mechanism. Whereas, the fraction of void occurrence is more in RE-PBR when the network is sparse because, it is hard to find forwarder node with high link quality along with the highest remaining battery and lower depth node. Moreover, the quantity decreases significantly as the density increases from 150–450. The reason of sudden decrease was the availability of more nodes in the transmission range of source node. As it is illustrated in Figure 8, RE-PBR only beats EnOR, while in other schemes, the mechanism of recovery is available which makes them more effective and efficient in terms of handling energy consumption.

#### 7.2.2. Depth Adjustment

**Figure 9:**
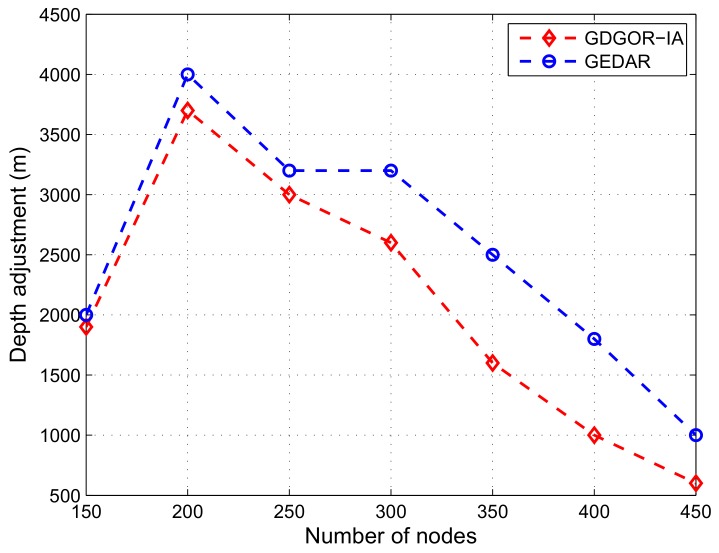
Depth adjustment plots.

At low network density, distance between void nodes is high. Figure 9 depicts the displacement of void nodes in GDGOR-IA and GEDAR. It can be seen that at node number 200, 15% of network nodes are void nodes. As node number in the network field increases, the displacement of void nodes decreases. This is because of increase in node density, the fraction of void nodes decreases as shown in Figure 8.

#### 7.2.3. PDR

**Figure 10:**
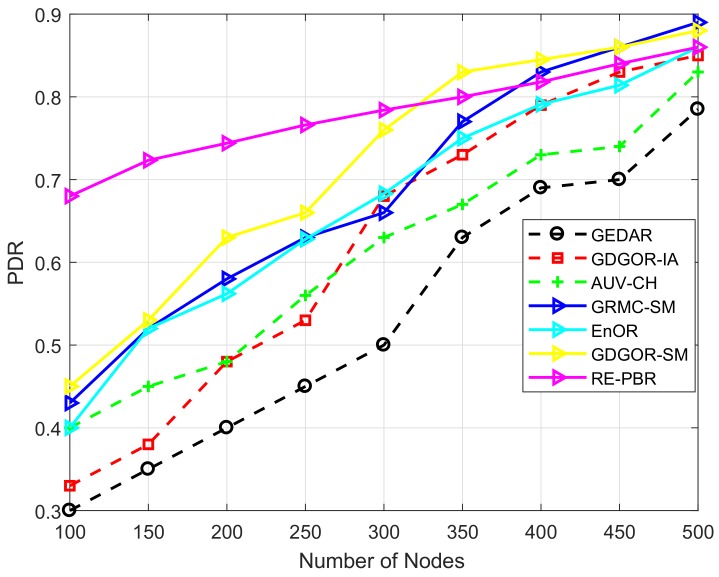
PDR plots.

The PDR of all schemes is monotonically increasing as depicted in Figure 10. However, the proposed work supersedes all the existing compared schemes because of the incorporation of sonobuoys mobility. Although all the three proposed schemes have opted void node recovery mechanism, cost associated with each scheme is different. At the beginning, GDGOR-IA performs same as GEDAR but the interference avoidance mechanism reduces the chance of data loss resulting in high PDR.

Initially, the PDR is very high of RE-PBR because of the consideration of link quality during the selection of forwarder node. The inclusion of link quality metric, enables reliable delivery of data packets at the destination as illustrated in Figure 10. The increase in PDR is gradual with the increase in node number because of consistent rotation of forwarder node, which avoids dramatic death of node. However, when node density reaches 350, the proposed schemes GDGOR-SM and GRMC-SM outperform RE-PBR because mobile sinks collect data directly from sensor nodes.

PDR in EnOR is very much high as compared to AUV-CH and even from proposed scheme, GDGOR-IA because of its ability to assign priorities to each node which ensures imbalance energy dissipation throughout the network operational time. However, the major reason of not beating all schemes is the absence of mobile sonobuoys due to which only data is delivered via multi-hopping. If void node occurs, then no mechanism is defined to recover data packet which results in data loss. While AUV-CH performs not well because of its ability to gather data from every node which takes time and gathers less data as compared to the proposed schemes.

#### 7.2.4. Energy Consumption

**Figure 11:**
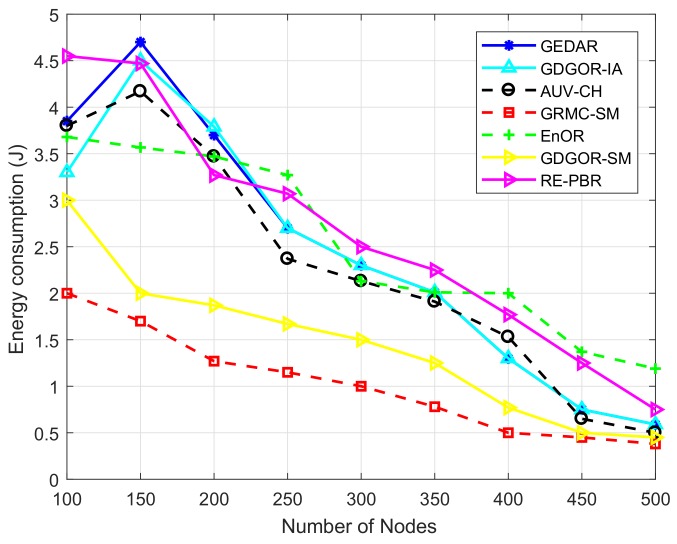
Energy consumption comparative plots.

The depletion of node battery is directly proportional to distance between transmitter node and receiver node. The energy utilization is presented in Figure 11 which clearly states that GRMC-SM outperforms rest of the compared schemes in the plot. Initially, the energy is 2 J at 100 nodes while as the density increases it goes down gradually to less than 0.5 J at 500 node number. The reason of this continuous fall down is that nodes start finding plenty of neighbors within the communication vicinity. As we said in the start of that discussion, energy consumption is directly related to distance, thus, when nodes find neighbors in the transmission range quite often and mobile sonobuoys continuously patrolling the acoustic environment than energy is significantly reduced by deploying more number of nodes. The pattern of energy dissipation in GDGOR-IA is the same, however, because of the consideration of interference, it needs to choose next hop with utmost care.

While, GDGOR-IA has more energy dissipation at node 100 however, it reduces as the node density increases but still has more energy than AUV-CH. In GEDAR and GDGOR-IA, energy consumption is mainly due to the depth adjustment for recovery purpose. At the beginning, fraction of void node is high in sparse network as shown in Figure 8. Hence, more energy consumption occurs due to large displacement of nodes on average to recover communication voids. The trend of energy consumption follows the same behaviour for GEDAR and GDGOR-IA when node number is below 250.

The AUV-CH and EnOR show moderate energy consumption from beginning till the node density 500. While, GEDAR has high energy consumption initially, but, it reduces suddenly after the node density increases from 150. The EnOR has minimum energy consumption 1.25 J when number of nodes are 500. Whereas, AUV-CH has slightly higher energy dissipation than GDGOR-IA as clearly depicted in Figure 11. Whereas, the dissipation of node battery is high in RE-PBR due to consistent rotation of relay node which helps in balancing energy, however, the involvement of more hops results in high energy consumption as compared to proposed schemes.

#### 7.2.5. End-To-End Delay

**Figure 12:**
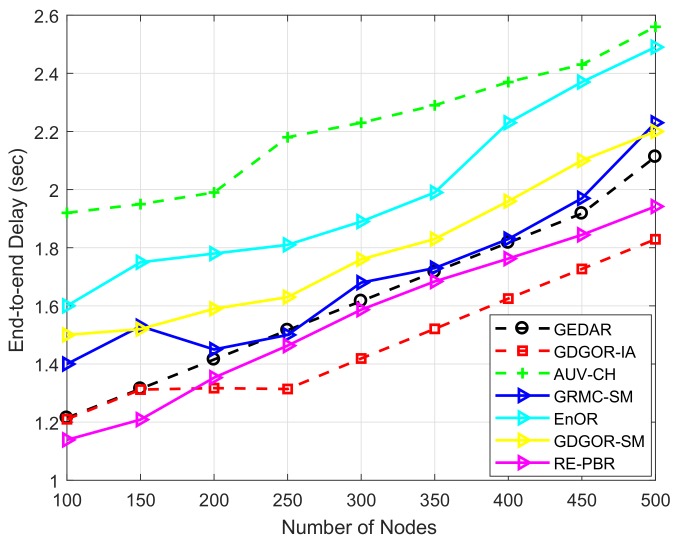
End to end delay plots.

In Figure 12, the end-to-end delay is consistently because of more number of nodes participate in communication when node density increases. Highest delay is experienced by AUV-CH due to data gathering from every node in its communication range, and the delay is 2.5 s at 500 node number. This delay is occurring because of high traffic load that results in to more number of transmissions. Whereas, EnOR has higher delay due to opportunistic forwarding in which time consumed at assigning priorities to each node in the forwarder set for avoiding immutable selection of each node towards the destination. This incorporates more delay in EnOR, however, lower than AUV-CH. While, delay in RE-PBR is less than all schemes throughout the network lifetime except GDGOR-IA. The reason of less delay than other schemes is, the selection of high quality link which mitigates the chances of retransmissions.

Whereas, GDGOR-IA bears the same delay as GEDAR in Figure 12. However, GDGOR-IA opts void hole avoidance mechanism along with interference avoidance in order to avoid communication voids and data loss. This incurs more delay due to several number of hops taken to by pass void holes. In GRMC-SM scheme, number of hops taken to deliver data to sinks is less while compared with other schemes because of mobile sonobuoys involvement for data gathering from acoustic nodes directly. Thus, reduced end to end delay is experienced in GRMC-SM and GDGOR-SM as shown in Figure 12. Performance analysis of GEDAR against proposed technique is given in Table 3.

#### 7.2.6. Performance Trade-Offs

From the simulation results, we can conclude that there is trade off between performance parameters. In GEDAR. GDGOR-IA scheme, achieves slightly better PDR is slightly high with 14% less delay in the network. This is due to the interference avoidance mechanism opted in the scheme that minimizes the delay caused due to the opportunistic routing opted in GDGOR-IA. GRMC-SM secures high PDR at low energy cost as compared with GRGOR-IA and GEDAR. While incorporating sink mobility in GDGOR-IA, energy cost associated with depth adjustment is diminished due to sink deployment in three dimensional volume for maximum coverage.

### 7.3. Observations of the Research

#### 7.3.1. Performance Analysis Based on Varying Traffic Loads

**Figure 13:**
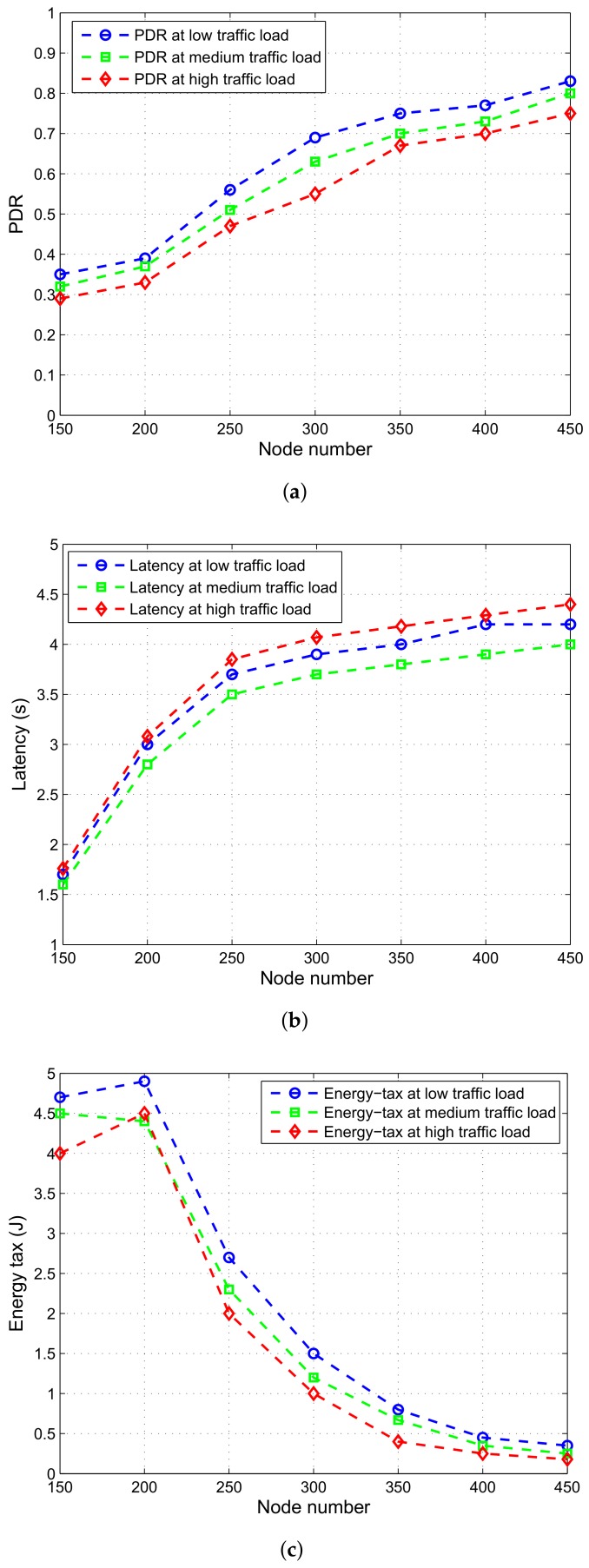
Performance parameters for GDGOR-IA. (**a**) PDR for GDGOR-IA; (**b**) latency for GDGOR-IA; (**c**) energy tax for GDGOR-IA.

To analyze the effect of traffic load in the network, we have carried out an analysis for GRGOR-IA routing scheme. At three different values of traffic load, we have evaluated performance parameters. In Figure 13a, PDR is high at medium packet size at 50 kbps data rate. PDR increases when node density is high, after the deployment of 350 nodes, it remains constant due to availability of node in the transmission range increases, however, few become potential forwarders. We have considered latency in Figure 13b that is high at high data packet size while considering same data rate for three data packet sizes. It is because, high traffic load incurs more delay overall in the transmission process. Whereas, latency incurred due to medium traffic load is less comparatively. Trend for energy consumption in Figure 13c follows same pattern for all three traffic loads. However, at high traffic load, energy consumption is high that is because of more energy consumption for high packet rate. Initially, energy consumption is more for medium traffic load while compared with low traffic load scenario. Later on, with the increase in node number, energy consumption stays same for medium and low traffic loads.

#### 7.3.2. Performance Analysis of GRMC-SM by Varying Number of Sinks

**Figure 14:**
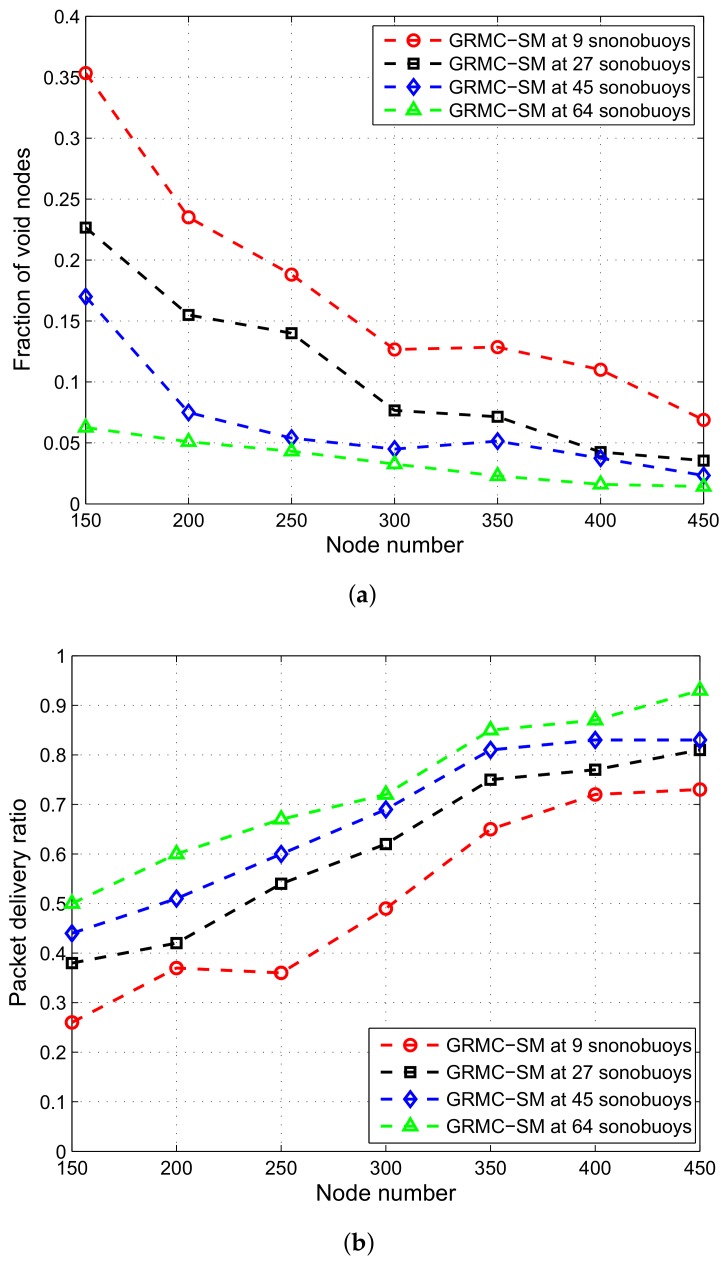

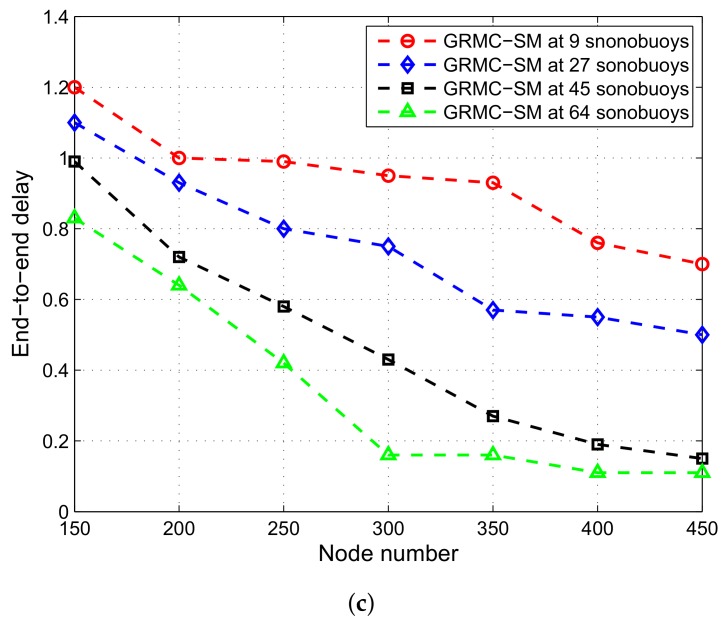
Performance parameters for GRMC-SM. (**a**) Fraction of void nodes under different number of sonobuoys; (**b**) PDR under different number of sonobuoys; (**c**) end to end delay under different number of sonobuoys.

To investigate the fraction of isolated nodes and their effect on PDR, we have conducted an analysis by varying sonobuoys from 9–64 sonobuoys. Void regions in the network are significantly reduced in GRMC-SM due to three dimensional deployment of sinks in the network region. Worst scenario is when number of sonobuoys are 9 and performance gets better with the increased number of sonobuoys. Because of increase in sonobuyoys number, the void regions and connectivity holes in the network are avoided. Hence, other performance parameters improve along with fraction of void nodes as shown in Figure 14. Considering the fact that only 5% nodes are in void region in case of 64 sonobuoys deployed in the network, we observe PDR gets higher in this scenario while compared with other scenarios. Average delay reduces due to more direct transmissions in 64 sonobuoys in the network. Anyhow, there are few costs associated with multi-sink architecture, specifically, when sinks are deployed in three dimensional field.

## 8. Conclusions

In this paper, the proposed schemes have performed collaborative tasks of routing data towards the destination while coping with communication voids. The proposed schemes exploit geographic information to route data greedily towards the sonobuoys. Three dimensional division has made network scalable and forwarding is directional because of selection of upstream nodes from the neighbor cube. In this way, hops taken to execute a complete transmission from sender node to sonobuoy has reduced significantly. Moreover, interference avoidance in GDGOR-IA helps in reduction of packet loss, thus it improves PDR. In GRMC-SM, controlled sink mobility considerably enhances network performance as compared to baseline schemes. Energy cost is significantly improved due to coping with communication voids by reducing fraction of void nodes. Consequently, these schemes provide efficient solution for reliable communication among the network nodes. Mathematical problem formulation using linear programming provides feasible solution for minimizing the consumption of energy, reducing average end-to-end delay and maximizing PDR. 

## Figures and Tables

**Table 1:** Nomenclature.

Symbols	Description
$C_{n}$	Number of logical cubes
$N_{n}$	Total nodes deployed
$S_{s}$	A set of sonobuoys
Λ	Flag to indicate that latest neighbor information
$F_{set}$	Potential forwarder nodes set
CC	Current cube
NC	Neighbor cube
TC	Target cube
$P_{BER}$	Bit error rate probability
$P_{CR}$	Probability of collision rate
ADV	Advancement towards destination
$T_{proc}$	Processing time at each node
$T_{p}$	Propagation delay
NADV	Normalized advancement towards destination
$MS_{n}$	Number of mobile sonobuoys deployed in the network
DFM	Data forwarding
$E_{TX}$	Transmission energy
$E_{RX}$	Receiving energy
$D_{i,j}$	Straight line distance from node *i* to node *j*
$E_{consumed}$	Energy consumed which includes transmission and reception energies
DR	Data rate
$E_{DA}$	Energy dissipated in data aggregation
$R_{c}$	Communication range of a node
$P_{TX}$	Transmission power for transmission data packet
$P_{RX}$	A power required to receive a data packet
PDR	Packet delivery ratio
$E_{th}$	The residual energy threshold which must be greater or equal than residual energies
$D_{DT}$	Delay occur in transmitting data packet directly
$D_{MHT}$	Delay occur in delivering data packet through multiple hops
$T_{hold}$	Time required to hold a packet

**Table 2:** Comparison of existing routing protocols.

Protocols	Void Handling	Geographic Information	Communication Overhead	Energy Efficiency	Delay
VBF [11]	No	Position information	High	Medium	High
DBR [13]	No	Depth information	Low	High	High
DS-DBR [14]	No	Depth information	Low	Medium	Low
H2-DAB [15]	No	Depth information	Low	High	Medium
RDBF [16]	No	Location information	High	High	Low
RMTG [17]	By pass void hole	Location information	High	Low	Medium
ARP [18]	Avoids void hole	Location information	High	High	Medium
DVRP [19]	No	Location information	Medium	High	Medium
VAPR [20]	Avoids void hole	Location information	Medium	Low	Medium
Hydrocast [21]	Depth recovery	Pressure information	High	Low	Medium

**Table 3:** Analysis of performance parameters against GEDAR.

Parameter	GDGOR-IA	GRMC-SM	GDGOR-SM
PDR (%)	4	7	3
Energy tax (%)	10	51	12
Latency (%)	16	−48	15

## References

[1] Akyildiz I.F., Pompili D., Melodia T. (2005). Underwater acoustic sensor networks: Research challenges. Ad Hoc Netw..

[2] Vasilescu I., Kotay K., Rus D., Dunbabin M., Corke P. Data collection, storage, and retrieval with an underwater sensor network. Proceedings of the 3rd International Conference on Embedded Networked Sensor Systems.

[3] Xu N., Rangwala S., Chintalapudi K.K., Ganesan D., Broad A., Govindan R., Estrin D. A wireless sensor network for structural monitoring. Proceedings of the 2nd International Conference on Embedded Networked Sensor Systems.

[4] Szewczyk R., Mainwaring A., Polastre J., Anderson J., Culler D. An analysis of a large scale habitat monitoring application. Proceedings of the 2nd International Conference on Embedded Networked Sensor Systems.

[5] Coutinho R.W.L., Boukerche A., Vieira L.F.M., Loureiro A.A.F. GEDAR: Geographic and opportunistic routing protocol with depth adjustment for mobile underwater sensor networks. Proceedings of the 2014 IEEE International Conference on Communications (ICC).

[6] Coutinho R.W., Boukerche A., Vieira L.F., Loureiro A.A. (2015). A novel void node recovery paradigm for long-term underwater sensor networks. Ad Hoc Netw..

[7] Coutinho R.W., Boukerche A., Vieira L.F., Loureiro A.A. (2016). Geographic and opportunistic routing for underwater sensor networks. IEEE Trans. Comput..

[8] Coutinho R.W., Boukerche A., Vieira L.F., Loureiro A.A. EnOR: Energy balancing routing protocol for underwater sensor networks. Proceedings of the 2017 IEEE International Conference on Communications (ICC).

[9] Liu M., Ji F., Guan Q., Yu H., Chen F., Wei G. On-surface wireless-assisted opportunistic routing for underwater sensor networks. Proceedings of the 11th ACM International Conference on Underwater Networks and Systems.

[10] Menon V.G., Prathap P.M.J. Comparative analysis of opportunistic routing protocols for underwater acoustic sensor networks. Proceedings of the International Conference on Emerging Technological Trends (ICETT).

[11] Kartha J.J., Jacob L. (2015). Delay and lifetime performance of underwater wireless sensor networks with mobile element based data collection. Int. J. Distrib. Sens. Netw..

[12] Maihofer C. (2004). A survey of geocast routing protocols. IEEE Commun. Surv. Tutor..

[13] Li Z., Yao N., Gao Q. (2014). Relative distance based forwarding protocol for underwater wireless networks. Int. J. Distrib. Sens. Netw..

[14] Dhurandher S.K., Obaidat M.S., Gupta M. A novel geocast technique with hole detection in underwater sensor networks. Proceedings of the 2010 IEEE/ACS International Conference on Computer Systems and Applications (AICCSA).

[15] Guo Z., Colombi G., Wang B., Cui J.H., Maggiorini D., Rossi G.P. Adaptive routing in underwater delay/disruption tolerant sensor networks. Proceedings of the Fifth Annual Conference on Wireless on Demand Network Systems and Services (WONS 2008).

[16] Yan H., Shi Z.J., Cui J.H. DBR: Depth-based routing for underwater sensor networks. Proceedings of the International Conference on Research in Networking.

[17] Wahid A., Lee S., Jeong H.J., Kim D. Eedbr: Energy-efficient depth-based routing protocol for underwater wireless sensor networks. Proceedings of the Advanced Computer Science and Information Technology.

[18] Javaid N., Jafri M.R., Ahmed S., Jamil M., Khan Z.A., Qasim U., Al-Saleh S.S. (2015). Delay-Sensitive Routing Schemes for Underwater Acoustic Sensor Networks. Int. J. Distrib. Sens. Netw..

[19] Ayaz M., Abdullah A., Faye I., Batira Y. (2012). An efficient dynamic addressing based routing protocol for underwater wireless sensor networks. Comput. Commun..

[20] Ali T., Jung L.T., Faye I. (2014). Diagonal and vertical routing protocol for underwater wireless sensor network. Procedia Soc. Behav. Sci..

[21] Xie P., Cui J.H., Lao L., Boavida F., Plagemann T., Stiller B., Westphal C., Monteiro E. (2006). VBF: Vector-based forwarding protocol for underwater sensor networks. NETWORKING 2006. Networking Technologies, Services, and Protocols; Performance of Computer and Communication Networks; Mobile and Wireless Communications Systems.

[22] Liang W., Luo J., Xu X. Prolonging network lifetime via a controlled mobile sink in wireless sensor networks. Proceedings of the 2010 IEEE Global Telecommunications Conference (GLOBECOM 2010).

[23] Baruah P., Urgaonkar R., Drishnamachari B. Learning-enforced time domain routing to mobile sinks in wireless sensor fields. Proceedings of the IEEE EmNets.

[24] Kumar V.N., Kumar M.S., Rajakumari J., Mohanarangan S. (2017). Opportunistic Void Avoidance Routing for Underwater Sensor Networks. Int. J. Sci. Res. Comput. Sci. Eng. Inf. Technol..

[25] Hsu C.C., Liu H.H., Gómez J.L.G., Chou C.F. (2015). Delay-sensitive opportunistic routing for underwater sensor networks. IEEE Sens. J..

[26] Noh Y., Lee U., Lee S., Wang P., Vieira L.F.M., Cui J., Gerla M., Kim K. (2016). Hydrocast: Pressure routing for underwater sensor networks. IEEE Trans. Veh. Technol..

[27] Lee U., Wang P., Noh Y., Vieira L.F.M., Gerla M., Cui J. Pressure routing for underwater sensor networks. Proceedings of the 2010 Proceedings IEEE INFOCOM.

[28] Khasawneh A., Latiff M.S.B.A., Chizari H. (2017). A reliable energy-efficient pressure-based routing protocol for underwater wireless sensor network. Wirel. Netw..

[29] Khan J.U., Cho H.S. (2015). A distributed data-gathering protocol using AUV in underwater sensor networks. Sensors.

[30] Erol M., Vieira L.F.M., Gerla M. Localization with Dive’N’Rise (DNR) beacons for underwater acoustic sensor networks. Proceedings of the Second Workshop on Underwater Networks.

[31] O’Rourke M., Basha E., Detweiler C. Multi-modal communications in underwater sensor networks using depth adjustment. Proceedings of the Seventh ACM International Conference on Underwater Networks and Systems.

[32] Yu Z., Xiao C., Zhou G. (2014). Multi-objectivization-based localization of underwater sensors using magnetometers. IEEE Sens. J..

[33] Kleerekoper A., Filer N. Revisiting blacklisting and justifying the unit disk graph model for energy-efficient position-based routing in wireless sensor networks. Proceedings of the 2012 IFIP Wireless Days (WD).

[34] Melodia T., Pompili D., Akyildiz I.F. Optimal local topology knowledge for energy efficient geographical routing in sensor networks. Proceedings of the Twenty-Third Annual Joint Conference of the IEEE Computer and Communications Societies (INFOCOM 2004).

[35] Yu H., Yao N., Liu J. (2015). An adaptive routing protocol in underwater sparse acoustic sensor networks. Ad Hoc Netw..

[36] Lee S., Bhattacharjee B., Banerjee S. Efficient geographic routing in multihop wireless networks. Proceedings of the 6th ACM International Symposium on Mobile Ad Hoc Networking and Computing.

[37] Xie P., Zhou Z., Peng Z., Yan H., Hu T., Cui J.H., Shi Z., Fei Y., Zhou S. Aqua-Sim: An NS-2 based simulator for underwater sensor networks. Proceedings of the MTS/IEEE Biloxi-Marine Technology for Our Future: Global and Local Challenges (OCEANS 2009).

